# Locomotion and hunting strategies of large vertebrate predators in pelagic waters

**DOI:** 10.1098/rstb.2025.0194

**Published:** 2026-10-15

**Authors:** Paolo Domenici, Federica Spina, Eve Jourdain

**Affiliations:** ^1^ IBF, CNR, Pisa, Province of Pisa, Italy; ^2^ Department of Biology, Ethology Unit, University of Pisa, Pisa, Province of Pisa, Italy; ^3^ Norwegian Orca Survey, Andenes, Norway; ^4^ Biosciences, University of Oslo Faculty of Mathematics and Natural Sciences, Oslo, Norway

**Keywords:** locomotion, predation, scaling, group hunting, pelagic zone

## Abstract

Many of the key dynamics that shape predator–prey interactions are governed by the physical and physiological principles which determine how animals move and perform in their environments. This opinion paper outlines the foundations underlying marine vertebrate predator–prey encounters within the context of pelagic habitats. Variables, such as maximum swimming speed, acceleration and manoeuvrability, play crucial roles in dictating the outcome of predator–prey encounters. In pelagic areas, sustainable swimming speed is an additional factor that can play an important role when chases occur. These locomotor traits are, in turn, influenced by body size, morphology and physiology. For predator–prey size ratio between 10 and 100, predators resort to a number of alternative strategies in order to overcome the elusiveness of the prey resulting from their higher manoeuvrability. These include pursuit leading to prey exhaustion, group hunting and the use of weapons. The framework of how physiological, physical and scaling rules affect pelagic predator–prey systems is a useful tool for investigating multi-predator feeding aggregations, where the relative success and potential competition between each predator species will be dictated by the potential strategies available to them within the scope of their performance limits.

This article is part of the theme issue ‘Multi-predator feeding aggregations in pelagic waters’.

## 1. Introduction

Within the context of predator–prey relationships, the study of multi-predator feeding aggregations (MPFAs) has emerged as a relatively recent area of research [1,2]. MPFAs represent complex, dynamic interactions in which multiple predator species simultaneously exploit a shared prey resource. These events can be observed in productive pelagic systems, where prey such as small schooling fish are driven to the surface by initial predators (e.g. dolphins), subsequently attracting a diverse assemblage of secondary predators, including seabirds, large teleosts, such as tunas and billfishes, and marine mammals such as pinnipeds [3]. Such aggregations provide a natural laboratory for examining how predators with differing morphologies, physiologies and foraging strategies interact both with one another and with their prey.

Despite increasing recognition of MPFAs, a mechanistic understanding of how species-specific traits shape predator roles and interactions within these events remains limited. In particular, there is a need to synthesize how locomotor performance, body size, thermal physiology (ectothermy versus endothermy) and the use of specialized structures (e.g. tails and bills) influence hunting strategies and success in a shared predation context. These factors are likely to vary across different phases of predation, such as pursuit, herding and capture, and may facilitate complementary or opportunistic interactions among predator species. For example, one species may benefit from prey aggregation or exhaustion caused by another, highlighting the importance of trait-mediated facilitation in these systems.

Here, we lay the foundation for a trait-based framework to evaluate predator performance within MPFAs in pelagic environments. Focusing on aquatic vertebrates, including fishes and marine mammals, we synthesize existing knowledge on the scaling of key locomotor traits and examine how these traits influence predator–prey dynamics across different stages of the hunting sequence. By integrating physiological, morphological and behavioural perspectives, this opinion paper aims to advance understanding of the functional roles of large pelagic predators and provide a basis for future research on their ecological interactions and implications for marine ecosystems.

Specifically, a list of points (hypotheses and questions) will be explored here where data are available and can be considered for future work in this field:

(i) the herding phase of the prey is likely to be carried out by a group of predators. One single predator is unlikely to be able to herd a full fish school to the surface;
(ii) the final phase (disruption of the prey group) is likely to be owing to collective predators, since ‘en masse’ attacks will create confusion in the prey;
(iii) ‘en masse’ attacks in the prey may be more likely to occur in predators with limited manoeuvrability. Predators with high manoeuvrability or with bodily weapons are likely to perform attacks as solitary, even though the group or predators may serve to constrain the group of prey;
(iv) within an MPFA, the occurrence of species with similar predation strategies (and body shapes/sizes) will increase competition. Alternatively, both predators may cause the prey school to be in a formation (e.g. dispersed) that benefits both species;
(v) within an MPFA, the occurrence of species with different predation strategies (and body shapes/sizes) will lead to behavioural niche separation. Alternatively, taxa of very different sizes and shapes (baleen whale versus teleosts, seabirds versus marine vertebrates) may prefer opposing prey school formations (e.g. compressed versus dispersed, respectively) and may therefore hinder each other's intake rates;
(vi) what combination of body shapes are predicted to cause attack strategies that lead to the greatest competitive interference – and why? and
(vii) what physiologies/morphologies preclude predators from particular stages of the hunt?

## 2. Scaling of unsteady locomotion

Historically, unsteady locomotion (i.e. accelerations and turning) has been considered crucial in predator–prey interactions [4,5]. This clearly applies to encounters in structurally complex habitats, where interactions are short lived because of the availability of refuges and hiding places. In pelagic habitats, other components may influence the outcome of a predator attack, and these will be discussed in §§3–5. Nevertheless, in both structurally complex and open (pelagic) habitats, the scaling of unsteady locomotion is fundamental to understand how predators and prey interact, because of the large difference in predator and prey sizes. Some of these issues have been reviewed by Domenici [6], Vogel [7] and Goldbogen & Cade [8]. Below, a summary of the main points, including additional recent papers, is given. This scaling framework also provides a mechanistic basis for predicting how differences in locomotor performance may translate into distinct foraging roles and interaction outcomes within MPFAs (points (v) and (vii)).

### (a) Speed

Maximum speed is a key factor in predator–prey interactions. Aquatic vertebrate predators generally swim faster than their smaller prey. Earlier work (e.g. [6]) focused mainly on fishes, while broader meta-analyses by Hirt *et al.* [9] and Sánchez-Rodríguez *et al.* [10] examined many aquatic species, revealing general size–speed patterns. Sánchez-Rodríguez *et al.* questioned some extreme values in Hirt's dataset, noting they relied on grey literature or indirect estimates, such as the often-cited 30 m s^−1^ for sailfish. More recent field and physiological data suggest sailfish reach about 8 m s^−1^ [11,12], and marine vertebrates probably do not exceed 10–15 m s^−1^ owing to cavitation risks [13]. Sánchez-Rodríguez *et al.*'s analyses excluding grey literature data show speed increases with body size up to approximately 1 m, then plateaus around 5–10 m s^−1^. Overall, evidence consistently indicates that small pelagic fishes (10–30 cm) are much slower than their larger predators (figure 1A).

**Figure 1.**
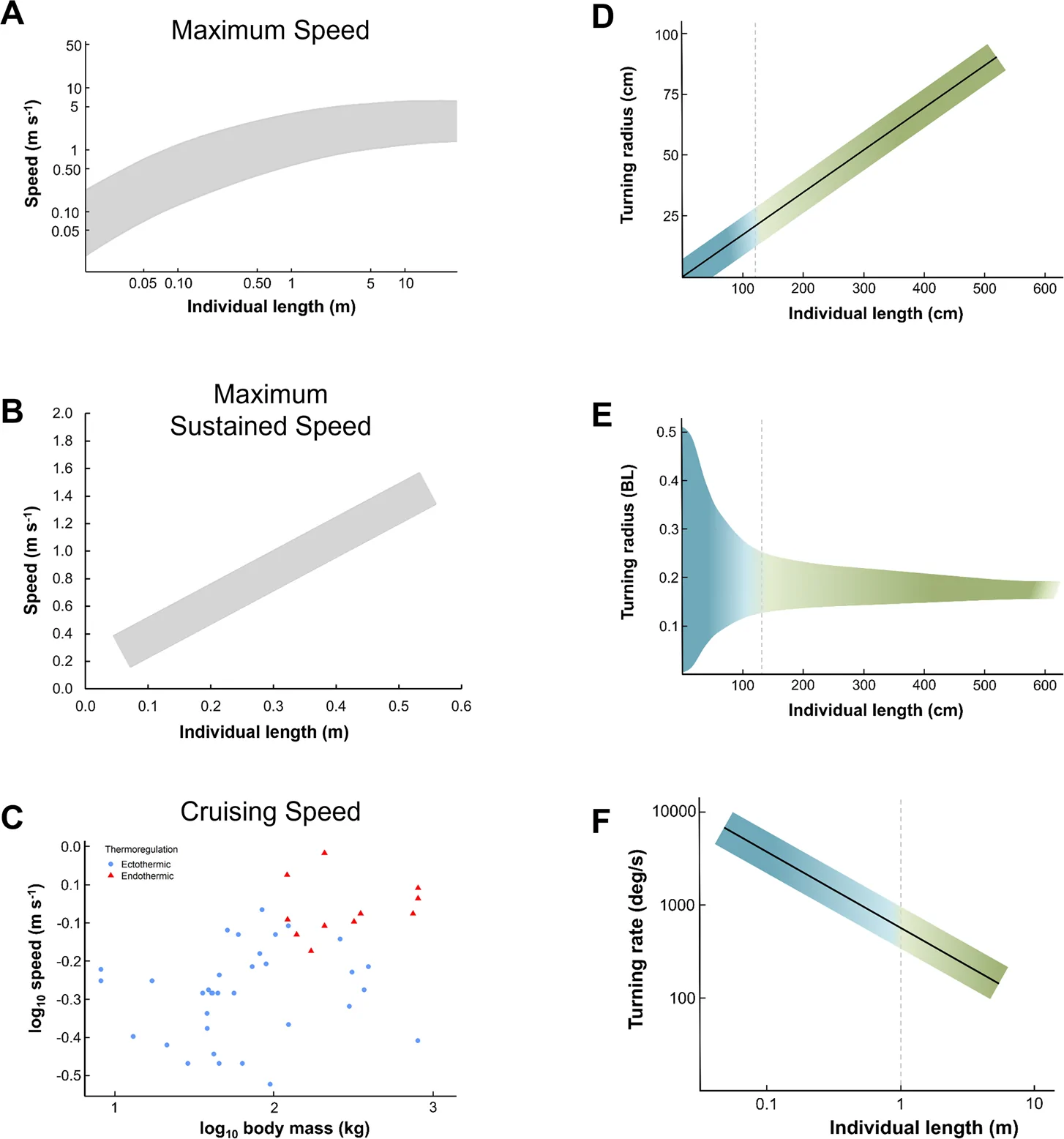
(A) Maximum speed versus length in aquatic animals (including agnathans, cartilaginous fishes, ray-finned fishes, lobe-finned fishes, amphibians, reptiles and mammals; redrawn based on Sánchez-Rodríguez *et al.* [10]). (B) Maximum sustained speed (*U*_ms_ in m s^−1^) versus length (*L* in m) in teleosts: *U*_ms_ = 0.15 + 2.4 *L* (*r*^2^ = 0.76), redrawn based on Videler [14]. (C) Cruising speed versus log_10_ of body mass in fishes with regional endothermy (red triangles) and fishes without it (blue circles) recorded in the wild, from Harding *et al.* [15]. (D) Turning radius (TRAD in cm) versus length (*L* in cm) in fishes (blue) and cetaceans (green) (TRAD = 0.17 *L* + 0.41 (*r*^2^ = 0.97), redrawn based on Domenici [6]. (E) Turning radius (in body lengths, BL) versus length in fishes and cetaceans, redrawn based on Domenici [6]. (F) Turning rate (TR, in degrees s^−1^) versus length (*L*, in cm) in fishes and cetaceans (logTR = −0.80 log*L* + 4.3; *r*^2^ = 0.92), redrawn based on Domenici [6].

Differences may also occur between the speeds of the various predators, based on their own size and physiological characteristics, and this may matter in terms of potential competition at a given foraging site. Indeed, such interspecific differences in locomotor performance are expected to influence competitive dynamics within MPFAs, particularly when species overlap in prey use but differ in their ability to access or exploit prey patches (points (b) and (f)). However, an important question that arises first is, how much does maximum speed matter for a predator to be able to catch its prey? In a structurally complex environment, where time to catch a prey is constrained by the availability of refuges, the maximum speed achievable within a given time is what matters most because prey can hide after a given time. Provided that such time is relatively short (i.e. a fraction of a second), previous work shows that large fishes are not faster than small fishes [6,16], in line with the fact that their acceleration is not superior (see below). By contrast, in pelagic waters, opportunities to catch prey are not constrained by the availability of refuges for the prey. Therefore, given the longer time available, predators could potentially reach their highest speed and catch the prey, if both were to swim in a straight line. However, this is a simplistic scenario because prey can turn swiftly, and therefore, speed in a straight line is only one of the many performance traits that matter. As we shall see, manoeuvrability can also play an important role in predator–prey interactions. In particular, trade-offs between speed and manoeuvrability are central to predicting whether predators rely on individual versus collective attack strategies (point (iii)) and how different morphologies may interact to generate either facilitation or interference (point (vi)).

In addition, considerations related to the effect of predator speed on the response of the prey need to be taken into account. Lunging at maximum speed does not necessarily pay off for a predator, because it has been shown that a high attack speed induces an early visual response of the prey [17]. This has been observed in laboratory experiments using real and predator models [17,18] and in field observations of pelagic predators [19]. Humpback whales attacking anchovies’ schools tend to swim at a submaximal speed (around 4.5 m s^−1^, [19], while they are capable of much higher speeds; table 1), thus minimizing the reaction distance of the prey with a stealthy type of approach [19]. This suggests that some predators may be constrained from performing high-speed solitary attacks without alerting prey, potentially favouring alternative strategies such as coordinated group attacks or stealth approaches (points (ii) and (iii)). In addition to predator speed, its shape (profile) is an important factor for minimizing the prey FID (i.e. flight initiation distance by the prey [55]). Predators with a tapered snout (e.g. sailfish) can approach at high speed without causing an early visual reaction in the prey [17]. This is because visual escape reactions are determined by the looming rate of the approaching threat, which is related to its speed and body diameter [17,18]. Similar effects were also shown in FIDs of the prey that are mediated by the lateral line, which increase with the size and speed of the approaching threat [56]. Such morphological adaptations illustrate how specific body shapes can mitigate sensory constraints imposed by prey detection, thereby influencing the type of attack strategy employed and the potential for overlap or complementarity with other predators (points (iii) and (vi)). In all cases, structurally complex habitats promote ambush in the predator, thus minimizing prey FID, a situation that cannot be replicated in MPFAs in pelagic waters. On the other hand, the scaling of another type of speed, that is maximum sustained speed (figure 1B), could be to the advantage of certain pelagic predators, as shown in §3.

**Table 1.** Previous work on performance traits and strategies related to predator–prey encounters in seven species of pelagic predators, some of which may co-occur during predation events.

species	maximum swimming speed	cruising speed	turning radius	turning rate	weapon	group hunting
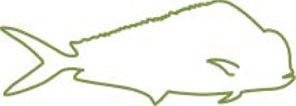 mahi-mahi *Coryphaena hippurus*	^a^**4 m s^−^^1^** (*L* = 0.69 m) [11];					
		**1.15 m s^−^^1^** (*L* = 0.31 m) [20]	**0.13 *L*** (*L* = 1.08 m) [21]			**✓** [22]
	**4.9 m s^−^^1^** (*L* = 0.20–0.35 m) [20]					
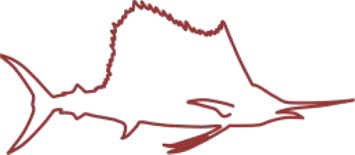 sailfish *Istiophorus platypterus*	^a^**8.3 m s^−^^1^** (*L* = 1.47 m) [11];					
		**1.9–2.3 m s^−^^1^** (*L* = 1.96–2.20 m) [12]		**575 deg s^−^^1^** (*L* = 1.8 m) [23]	**✓** [23]	**✓** [24]
	**5.2–7.0 m s^−^^1^** (*L* = 1.96–2.20 m) [12]					
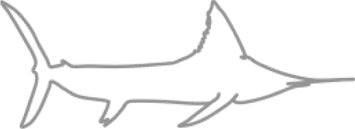 striped marlin *Kajikia audax*		**1.9 m s^−^^1^** (*L* = 1.80 m) [25]			**✓** [26]	**✓** [27]
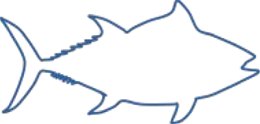 tuna^b^ *Thunnus* spp.		**0.9-2.6 m s^−^^1^** (*L* = 0.75–0.94 m) [29];	**0.47 *L*** (*L* = 0.30 m) [31];			
	^a^**15 m s^−^^1^** (*L* = 2.26 m) [28]			**302 deg s^−^^1^** (*L* = 1.37 m) [32]		**✓** [3,33]
		^a^**1.85 m s^−^^1^** (*L* = 0.5 m) [30]	**0.38 *L*** (*L* = 1.37 m) [32]			
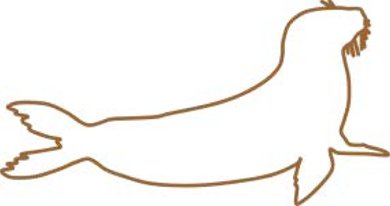 sea lion^b^			**0.11–0.19 *L*** (*L* = 1.72–1.89 m) [35];	**514–599 deg s^−^^1^** (*L* = 1.72–1.89 m) [35];		
		^c^**2.0 m s^−^^1^** (*L* = NA) [34]				**✓** [37]
			**0.13–0.16 *L*** (*L* = 1.82–2.24 m) [36]	**353–541 deg s^−^^1^** (*L* = 1.82–2.24 m) [36]		
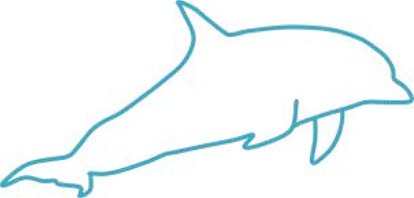 dolphin^b^	**8–8.2 m s^−^^1^** (*L* = 1.83–2.95 m) [38]	^c^**3.1 m s^−^^1^** (*L* = NA) [34];	**0.32 *L*** (*L* = NA) [40]	**202 deg s^−^^1^** (*L* = NA) [40]	**✓** [41]	**✓** [3,42,43]
		^c^**2–3 m s^−^^1^** (*L* = NA) [39]				
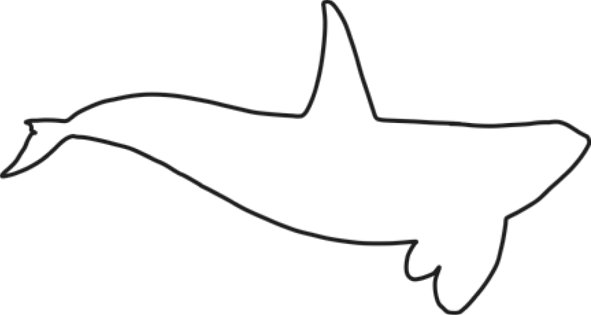 killer whale *Orcinus orca*	^c^**12.5 m s^−^^1^** (*L* = NA) [34];					
		**3.7 m s^−^^1^** (*L* = NA) [45]	**0.41 *L*** (*L* = NA) [40]	**111 deg s^−^^1^** (*L* = NA) [40]	**✓** [46]	**✓** [47–49]
	**15.5 m s^−^^1^** (*L* = NA) [44]					
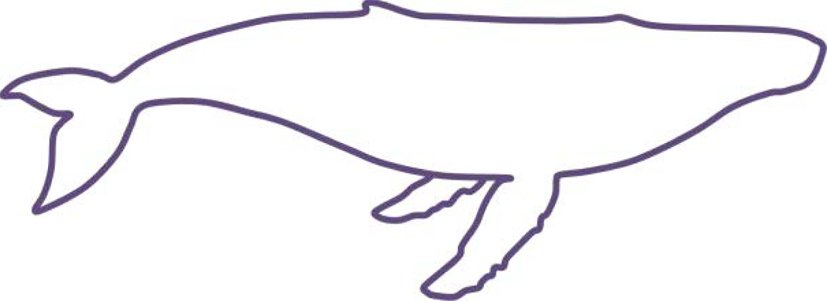 humpback whale *Megaptera novaeangliae*	**8.2 m s^−^^1^** (*L* = 14.7 m) [50]	^c^**3 m s^−^^1^** (*L* = 12.2m) [51]	**0.14 *L*** (*L* = NA) [52]		**✓** [53]	**✓** [54]

*Notes: L* = body length; NA data not available.

^a^ Indicates theoretical estimates.

^b^ Indicates data on various species.

^c^ Estimated from figure.

Although ambush predation is likely to be less feasible in pelagic environments owing to the absence of physical structures such as vegetation or rocks that provide concealment, alternative mechanisms may still facilitate stealth-based attacks in the open ocean as suggested for humpback whales and sailfish. For instance, predators may exploit attack geometry, approaching prey from below where they are less detectable against downwelling light or use forms of camouflage that minimize visual contrast with the background [57]. Despite this, direct evidence for ambush behaviour in open water systems remains limited, with most documented cases involving sharks or prey adaptations such as countershading. This apparent scarcity may reflect the inherent challenges of observing and experimentally manipulating predator–prey interactions in the open ocean, rather than a true absence of such strategies. Notably, dynamic coloration and patterning, such as vertical bars or stripes, are widespread among pelagic teleosts, including taxa like sailfish, marlin, wahoo, tuna and Spanish mackerel, and may play a functional role in transient camouflage and signalling during predator attacks.

### (b) Acceleration

Acceleration is an important factor in predator–prey interactions, influencing escape success [5]. Studies show that body morphology, such as a large tail and a deep posterior body depth, enhances acceleration [58]. However, clear scaling patterns across taxa are lacking, partly because small animals are studied in laboratories with high-speed video (based on double differentiation of distance data), while larger animals are measured in the field with accelerometers. Data suggest small fishes (5–20 cm) may accelerate faster (50–150 m s^−^²) than larger fishes or marine mammals (<10 m s^−^²). These differences in acceleration capacity may constrain the ability of larger predators to capture agile prey individually, thereby promoting reliance on collective hunting or alternative strategies such as the use of weapons (points (ii), (iii) and (vii)). Furthermore, Segre *et al.* [59] found that as body size increases in baleen whales, absolute acceleration and manoeuvring performance decrease. However, overall variability in performance measures across species of vertebrates is high, and methodological differences probably bias results. Consequently, the possibility that higher acceleration consistently benefits prey needs to be taken with caution, as current evidence is confounded by measurement inconsistencies and variability.

### (c) Manoeuvrability: turning radius

Turning radius, the circle formed by an animal's centre of mass while turning, is usually 5–20% of body length in aquatic vertebrates. Hence, small prey (10 cm) can turn within approximately 1 cm, while large predators (3 m) turn within approximately 30 cm, allowing prey potential escape. Size largely determines turning radius, but morphology and flexibility also matter: the turning radius of flexible species (e.g. angelfish and some sharks) is as small as 7% of body length, while that of rigid fish (e.g. yellowfin and bluefin tuna) exceeds 20% (figure 1D,E). This size-dependent constraint on turning performance is probably a key driver of the need for cooperative or sequential hunting strategies in some larger predators (points (i) and (ii)), as well as a determinant of which species can effectively engage in close-range attack phases (point (vii)).

In addition, fins and other surfaces can aid in manoeuvrability by reducing the turning radius, as observed from small fishes [60] to humpback whales [61]. This is also the case for sea lions (*Zalophus californianus*) that possess large flippers and a flexible design in terms of body and neck agility [62,63]. During unsteady manoeuvres, mahi-mahi fully extend their median fins, resulting in a tight turning radius compared to typical pelagic fishes, such as rigid-bodied tuna ([21]; table 1). Teleost species tend to show more variability in their relative turning radius than marine mammals, possibly owing to their wide variety of morphologies ([6]; see figure 1E). An interesting example is that of MPFA on small pelagic schooling prey off Baja California, where both sea lions and teleosts such as striped marlin and tuna may feed on the same patch. Here, the manoeuvrability of the sea lions is expected to be higher than that of the tuna (based on table 1) and possibly of the striped marlin, and this may be an important factor in determining their different hunting strategies (dashing versus manoeuvring around the prey; [64]). In addition, if the attacking predator (or a given predator species) can reposition itself quickly for another attack with a fast and sharp turn, it may be able to partially monopolize access to the prey school, increasing the chance for interference competition. The observed intraspecific differences provide a clear example of how morphological variation can lead to behavioural niche partitioning within MPFAs (point (v)), while also potentially reducing or enhancing competitive overlap depending on how prey are structured by each predator (point (iv)).

### (d) Manoeuvrability: turning rate

Turning rate, measured as the angle an animal turns within a given time, is size dependent: small animals turn faster than large ones ([6,65]; figure 1F). A 10 cm fish can make a 90° turn in approximately 30 ms, while a 3 m predator takes approximately 0.4 s, giving prey a potential escape advantage. Turning rate scales are in line with the scaling of tail beat frequency and muscle contraction time [6] and is also influenced by species-specific morphology and fin use. In addition, large predators can compensate for their turning handicap when attacking small and elusive prey by taking advantage of certain morphological or behavioural features. This is the case of the sailfish's large dorsal fin for enhancing attack stability or when using alternative behavioural strategies (e.g. group hunting and the use of weapons), as discussed below (§§4 and 5). These compensatory strategies highlight how limitations in manoeuvrability can favour collective attacks or the use of weapons (points (iii) and (v)), reinforcing the idea that different predator types contribute distinct functional roles within MPFAs (point (vii)).

## 3. Predator–prey length ratios

Most unsteady swimming abilities (except speed) generally benefit prey. Predator–prey size ratios help predict attack strategies [66]. When prey are relatively large (ratio < 10; [67]), their agility offers limited escape advantage, and predators typically use whole-body attacks [6,66,68]. When predators are vastly larger (ratio > 100), prey are treated as bulk ‘food units’ and consumed via methods like filter feeding, as their evasiveness becomes ineffective [66]. At intermediate ratios (10–100), prey size provides some advantage, forcing predators to adopt alternative strategies in pelagic settings, such as exhausting pursuit, group hunting or using specialized weapons, as discussed below. This size-dependent shift in strategy supports the prediction that collective herding (point (i)) and coordinated disruption (point (ii)) become increasingly important when prey are small and agile relative to their predators. These different strategies will be discussed below, with a focus on predator–prey interactions in pelagic habitats: pursuit leading to prey exhaustion, group hunting and the use of weapons (figure 2).

**Figure 2.**
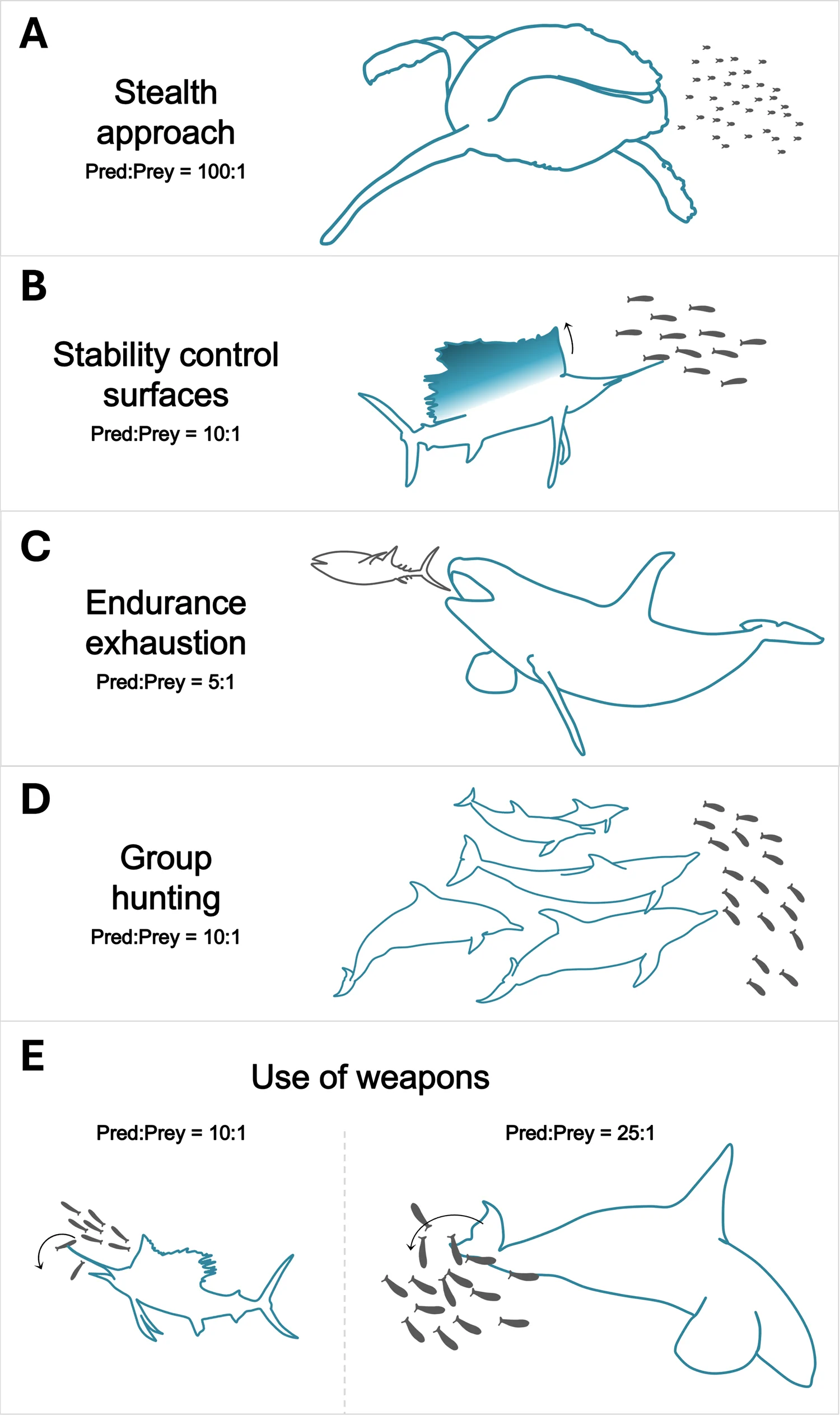
Alternative strategies used by large vertebrate predators to overcome the elusiveness of their smaller prey in pelagic habitats. Indicative predator : prey length ratios (pred : prey) are reported in each panel. See text for details.

## 4. Endurance: an additional locomotor trait that matters in predator–prey interactions in pelagic waters

Unlike coastal or structurally complex habitats where most early predator–prey studies have been conducted in laboratories with species like pike or trout, pelagic environments lack refuges, limiting sit-and-wait strategies and hiding places for the prey [69,70]; hence, repeated attacks on the prey (individual or school) can be made by a single or a group of predators. This flexibility suggests that both solitary and collective strategies may operate within the same system, with their relative importance depending on predator traits and environmental conditions (points (i), (ii) and (vii)). In complex habitats, prey can escape into cover after an attack, but in pelagic waters, continuous swimming performance and other traits beyond unsteady locomotion may determine predator–prey outcomes, requiring a revised perspective on which traits matter in these open water interactions.

### (a) Endurance exhaustion of the prey

Owing to the absence of refuges, long-duration pursuits [71–73] can occur in open waters, and therefore, sustained swimming abilities (e.g. maximum aerobic speed) may be a major determinant of whether or not a prey will avoid being captured. As in the case of maximum (burst) swimming speed (figure 1A), sustained swimming speed increases with size (figure 1B; [14]). Data on the sustainable speeds of large marine animals are not easy to obtain in the field. It is possible that their maximum aerobic speed also plateaus at a large size like burst swimming, since the sustained speed of humpback whales is similar to those of killer whales and dolphins (table 1). Since the predators’ sustained speed is probably higher than those of their smaller prey, a large predator can chase its prey to exhaustion, at which point it will be easy to catch. Such endurance-based strategies highlight how physiological capacity can determine participation in specific hunting stages, particularly prolonged pursuit (point (vii)), and may confer advantages over other predators within MPFAs (point (iv)). While this type of endurance exhaustion technique is relatively well known in terrestrial vertebrates [66], less is known about marine species, largely owing to difficulties in tracking these types of interactions over long periods and distances. Among the examples known in marine habitats are those of killer whales chasing bluefin tuna near the tuna's maximum aerobic speed, which results in capture after about 30 min ([45]; figure 2), and killer whales chasing false killer whales to exhaustion [74]. Other situations in which the hunting behaviour of the predator causes the prey to swimming near their maximum sustained speed were reported: sailfish pursuing sardines [23], striped marlin chasing sardines [75] and killer whales surrounding herring [46]. In addition, when the schooling prey is packed in high densities, their fatigue may also be exacerbated by hypoxic conditions [46,48,76] which may result in poorer ability to react to predator strikes. We predict that new technologies, such as drones and biologgers, will unveil more examples of endurance exhaustion of prey in pelagic environments, than the ones known thus far.

### (b) Ecto- versus endothermic predators

Because sustained swimming is one of the strategies large predators may use in pelagic environments to exhaust their prey, it becomes relevant to identify the physiological characteristics that affect this type of performance. With respect to the main species we are concerned with, that is large predatory fishes and marine mammals, a major determinant of sustained swimming speed is whether the predator is ecto- or endotherm, in addition to their size. Endothermy allows marine vertebrates to keep a high metabolic rate and to operate their red (aerobic) muscles at relatively high temperatures even in cold waters [77]. This implies that their speed can be maintained even at low temperatures, and Grady *et al.* [78] suggested that predation on ectothermic prey (such as pelagic schooling fishes) is probably easier in cold waters, resulting in a high abundance of large endothermic predators in polar areas.

Within teleosts, certain species (such as tuna and billfishes) are regionally endothermic, and previous work has shown that their cruising speed is higher than that of ectotherm teleosts ([15]; figure 1C). This difference has a number of implications: (i) the higher sustained speed of endotherms may be advantageous when chasing the prey to exhaustion; (ii) endotherm species may have a higher prey encounter rate [79]; and (iii) faster species (such as endotherms) may have a higher sensory performance for identifying and capturing prey as a result of higher visual resolution [80,81]. Cranial endothermy, as found in tuna and billfishes for the brain and eye region, may enhance these predators’ ability to visually detect their moving prey [82]. As a general rule, therefore, size and endothermy increase the continuous swimming performance of predators and may give a competitive advantage over smaller and ectothermic predators when involved in a long chase.

In addition to their role during active pursuit, physiological traits related to locomotion and the sensory system are also likely to influence earlier stages of the predation sequence, particularly prey encounter and patch location. In pelagic systems, prey are often highly abundant yet heterogeneously distributed, requiring predators to travel efficiently between spatially discrete resource patches. Under such conditions, endothermy and elevated sustained swimming speeds may enhance not only chase performance but also the rate at which predators encounter prey aggregations. This suggests that the advantages conferred by endothermy extend beyond the final stages of prey capture, to encompass a broader portion of the foraging process, including search efficiency and the ability to exploit ephemeral prey fields.

At the same time, pelagic predators are likely to face important trade-offs between efficient long distance locomotion and manoeuvrability, the latter being critical during the terminal phases of predator–prey interactions. These trade-offs provide a key mechanistic explanation for why less manoeuvrable predators may rely more heavily on collective ‘en masse’ attacks (point (ii)), while also predicting which combinations of predator traits are likely to generate competitive interference or facilitation (point (vi)). Morphological and physiological adaptations that favour sustained, energetically efficient swimming—such as streamlined body shapes and high aspect ratio lunate fins [17,83]—may impose constraints on turning performance and fine-scale control. As a result, species specialized for cruising may be less effective in close-range, highly manoeuvrable chases, potentially favouring alternative strategies such as group foraging ‘en masse’ as in the case of tuna [3]. These trade-offs may ultimately contribute to the diversification of feeding strategies observed among pelagic predators, reflecting different solutions to balancing the demands of searching, pursuing and capturing prey in the pelagic environment.

## 5. Group hunting

Cooperation among predators can lead to higher efficiency in prey capture and has been discussed in previous reviews and meta-analyses [66,84]. Here, we briefly outline relevant examples of cooperation that appear to be particularly effective in pelagic settings. Indeed, where the prey is not confined within physical barriers, cooperation among predators can be useful to enhance capture once the prey are surrounded and trapped between the encircling predators and the water surface. Hansen and colleagues [66, p. 1696] suggested that group hunting can enhance prey capture through three phases: ‘exhaustive pursuit and ambush; herding and condensing prey; and prey isolation and containment’. In pelagic systems, such cooperation is particularly relevant for overcoming the difficulty of constraining mobile prey, supporting the hypothesis that herding is primarily a collective process (point (i)). The type of interactions among group members while attacking prey can also be influenced by inter-individual bonds, with various levels of sociality, dependence, resource sharing, specialization and communication [84]. Generally speaking, smaller predators appear more likely to hunt in groups as they require a smaller proportion of the school of prey, and they may also group for defence against larger predators.

A notable example of cooperation that allows predation of otherwise elusive prey in the pelagic environment is that of Galapagos sea lions (*Zalophus wollebaeki*) herding amberstripe scad (*Decapterus muroadsi*) from a relatively open area to a coastal cove that acts as a ‘trap’ for the prey [37]. Here, prey were originally in a pelagic area, and that is where herding occurs, which ultimately leads to trapping in a coastal area. Previous work suggests that the predators may show complementary behaviours in herding the prey. Benoit-Bird & Au [43] showed that spinner dolphins foraged in coordinated groups in a pelagic environment, as a result of herding and concentrating the prey using group manoeuvres based on strict geometry and timing. This phase is central to enabling subsequent attack stages, reinforcing the idea that collective herding is a prerequisite for efficient prey exploitation in many pelagic systems (point (i)). Dusky dolphins have also been shown to herd prey to the surface [85], and killer whales can trap herring to the surface from a depth of about 160 m [86,87]. The cooperation achieved through various coordinated manoeuvres shows that in some cases group members take different roles. For example, in bottlenose dolphins, an individual was observed to consistently herd the prey towards a group of ‘barrier’ individuals [42]. In addition to these examples of collaborative predation involving mainly marine mammals, work on teleosts (sailfish) has shown that group hunting can provide an advantage to the predators even in the absence of higher social organization, coordinated attacks or individual roles in the hunt [24]. This is because more prey are injured by the bill slashes of the sailfish than are caught, facilitating prey capture in the whole group. This mechanism represents a form of facilitation, where the actions of one predator increase prey vulnerability for others, directly relevant to predictions about positive interactions within MPFAs (point (iv)). Clearly, group hunting with any level of interaction (or absence thereof), among predators, can be advantageous when attacking a group of small, elusive prey in a pelagic setting. In addition, herding/dispersing the prey by one species of predator may have a direct effect on the feeding success of other incoming predators (point (iii)). For example, herding herring schools by killer whales may facilitate lunge predation by humpback whales [88], while the isolation of prey as a result of sailfish attacks may facilitate prey capture by seabirds.

## 6. The use of weapons in large vertebrate predators

Certain pelagic predators have evolved a foraging strategy that circumvents the issue of their lower unsteady performance and manoeuvrability associated with their large relative size compared to their prey, by using weapons. This has been observed in tail whipping by thresher sharks [89], tail slapping by killer whales [46] and other delphinids [41], bill slashing in billfishes [23] and tusk-hitting by narwhals [90]. This illustrates how physiological and biomechanical constraints can be offset by alternative strategies, determining which predators can participate in specific hunting stages (point (vii)) and how they interact with other predators (point (vi)). From a motion performance perspective, the ‘weapon’ becomes the threat, and its acceleration was recorded to be much higher than that of the whole body of the predator and in line with the acceleration ability of their small prey [23,46]. For both bill slashing in sailfish and tail slapping in killer whales, based on the speed and acceleration of their ‘weapon’, the estimated maximum swimming performance and the reaction time of the prey, the latter are unlikely to be able to avoid the blow with which they are hit [23,46].

All of these examples are particularly effective when attacking schooling prey, as they typically follow an initial phase during which the predators are concentrating on the prey. This sequential structure strongly supports the hypothesis that collective herding precedes and enables effective disruption of prey (points (i) and (ii)). In pelagic waters of northern Norway, killer whales herd schooling herring to the surface and then surround them by using ‘carousel feeding’, during which killer whales show their white undersides towards the school, which was suggested to concentrate the prey [48]. Killer whales then strike the tightly packed herring school with the underside of their fluke, stunning up to 30–40 individuals with a single slap [46]. Given the feeding requirement of a typical range of killer whale size (estimated 285–578 herring d^−1^; [91]) and a realistic yield of 16 herring per tail slap [46], killer whales could get their daily food requirement with about 18–36 tail slaps, which is an estimate that appears realistic given the frequency of tail slap observed in the field for killer whale groups of 2–15 individuals (0.55 min^−1^ [49]; 1.47 min^−1^ [46]). In shallower coastal areas of northern Norway, killer whales instead operate in pairs, swimming sideways on either side of a herring school while facing each other's ventral side. One of the killer whales acts as a helper by concentrating the prey, while the other killer whale slaps the herring. The efficiency of this strategy is confirmed by the feeding time of such pairs, which is twice as long compared to when, occasionally, killer whales tail slap herring without a partner [49]. Such role differentiation provides evidence for functional specialization within predator groups, consistent with predictions of behavioural niche partitioning and facilitation (points (iv) and (v)).

In certain large vertebrate predators, other parts of the body can be used to increase the efficiency of this type of ‘weapon’ hunting. Sailfish typically first insert their bill into a school of fish and subsequently slash through the school [23]. Field observations have shown that their prey (sardines) do not react to the insertion of the bill into the school, so that the bill slash motion can be effective in hitting the prey nearby. This is possibly owing to a number of features of the bill. It is known that predators with narrow profiles can approach prey without causing a visual escape response because of the low level of looming rate of the threat [17,18]. In addition, the potential disturbance of the bill when inserted in the school is minimized by other factors. While, when cruising, the bill of sailfish shows a yaw motion of about +7° on each side, such sideways motion is largely dampened (< 1°) when the bill is inserted into a school, as a result of the extension of the dorsal and pelvic fins. These fin extensions serve to maximize ‘control surfaces’ ([92]; figure 2) and therefore minimize motion disturbance to the prey allowing for a stealth approach [12,23]. Disturbance minimization of the prey may also be related to the denticles present on the bill of sailfish, which, in addition to being an abrasive surface to damage the prey [24], may also minimize the hydrodynamic disturbance by interacting with the flow. Another billfish species, the striped marlin, also uses the bill as a weapon, but in a different way than sailfish [26]. Their bill is shorter and marlin does not possess a sail that helps dampen any side motion. Therefore, instead of inserting the bill into a school like sailfish do, striped marlin most often tend to dash in line towards the school and grab the prey after ‘tapping’ it with the bill, a strategy that has also been observed, though seldom, in sailfish [26]. The differences in the main strategy used by striped marlin and sailfish may therefore be associated to their different bill types and other morphological features such as the sail in sailfish and their lateral compression which facilitates manoeuvrability as needed at short distance from the prey which is how sailfish operate when attacking [26]. In addition to the extension of the sail, the extended pelvic fins may increase the overall accuracy of the manoeuvres of the sailfish bill after its insertion into the school of prey, such as tapping and slashing. Furthermore, the sail may also serve to resist lateral forces in the direction opposite to the slash, hence enhancing the effectiveness of the slash itself. The extension of the sail was also observed prior to the insertion of the bill into schools of prey; this may have other additional functions such as acting as a ‘herding’ device to concentrate schooling prey [23], similarly to the white underbelly of killer whales [48].

Much like the dorsal fin of sailfish, other predators have morphological structures that aid in predation even if they are not used as direct weapons that injure the prey. Such structures can help increase the efficiency of predation by limiting the elusiveness of their small, agile prey. An example is the large fins of the humpback whales. Kosma *et al.* [53] and McMillan *et al.* [93] show that humpback whales can use their fins to herd their prey or flick prey into its mouth, respectively. Kosma and colleagues [53] suggest that the humpback's fins can be used in three ways to herd the prey: (i) as a physical barrier, (ii) causing flow that guides prey towards the buccal cavity of the predator, and (iii) to reflect light through the central side (much like the underside of killer whales described above). Cade *et al*. [19] estimate that the use of pectoral fins may increase capture rates from 20% to 50% in lunging humpback whales. In addition to using their bodily weapons, as in the case of their pectoral fins, humpback whales can also take advantage of other ‘external’ foraging tools, such as the bubble net traps they produce with their blowholes while swimming in a circular path [94–96]. The creation of curtains of bubbles provides an advantage to the predator by concentrating the prey within the curtain. This way humpback whales can overcome the prey elusiveness [54], and this strategy was shown to be coupled with their relatively high predator manoeuvrability for an animal of that size, as a result of large pectoral flippers [52].

Importantly, weaponry may play a critical role in the dynamics of MPFAs by influencing prey accessibility across species. Prey injured or stunned through the use of weaponry are not only available to the individual or species using the body weapon but also to associated predators in the MPFA, such as birds or smaller teleosts that capitalize on stunned pelagic prey. This represents a key mechanism of interspecific facilitation within MPFAs, whereby one species enhances prey accessibility for others, potentially promoting aggregation formation (point (iv)). Furthermore, when weaponry is linked to prey herding or condensing behaviours, it may enhance the overall feeding efficiency of co-occurring predators. In some cases, the use of weapons by predators could even act as a primary factor driving the initial formation of MPFAs, with the aggregation forming around the opportunities created by prey manipulation. Considering the interplay between weaponry, prey behaviour and interspecific facilitation provides insight into the mechanisms structuring predator assemblages and the emergence of cooperative or opportunistic feeding interactions.

## 7. Aerial predator strategies

The presence of birds of various species is typically found in many MPFAs where they feed on concentrated fish schools [97]. Early work by Hoffman *et al.* [98] grouped such feeding associations into three types based on size, duration and type of food source. Type I flocks are small and short lived over tightly clustered prey, while type II flocks are large and longer-lasting over more dispersed prey. Type III flocks form where prey is concentrated by ocean processes like downwelling. Birds within these flocks fall into functional groups: catalysts that locate and signal food, divers that hunt underwater, kleptoparasites that steal prey and suppressors that reduce prey availability for others through interference. Camphyusen & Webb [99] further characterized MPFAs in the North Sea which vary in species richness depending on their context. Those around fishing vessels tend to have low diversity, while those linked to cetaceans show moderate diversity, and those over natural fish shoals have the highest richness.

Marine birds exhibit a diverse range of foraging strategies, each closely linked to their morphology and ecological niche. Seagulls and terns are opportunistic feeders that primarily capture fish near the surface without diving. Typically, these species take advantage of prey driven upwards by predatory fish, using their low flight patterns, strong wings and robust beaks to quickly seize accessible prey [100,101]. Frigate birds employ a different tactic, relying on surface feeding or kleptoparasitism—stealing prey from other birds. They do not dive, but their long, narrow wings and lightweight bodies make them exceptional aerial hunters, while their hooked beaks help them grasp prey mid-flight [102]. Gannets, by contrast, are highly specialized for plunge diving. Their dives can then be followed by pursuing fish below the surface [103]. Their streamlined bodies and sharp beaks reduce drag [104], while specialized cervical anatomy and pressure-dissipative feathers protect them from the impact of high-speed entry into the water [105]. Pelicans adopt yet another approach, using partial dives or scooping fish from the surface with their large gular pouches. They often operate in shallow waters, combining swimming and dipping behaviours to capture prey efficiently, and their foraging behaviour may involve some degree of cooperation [106,107]. Potentially, bird species that pursue their prey below the surface are more likely to interfere directly with the motion of large vertebrate predators such as billfishes and cetaceans. However, even bird species that only operate at the surface by picking up injured prey can create competition in terms of prey availability for other aquatic predators (points (iv) and (v)). Despite clear differences in diving ability and feeding techniques—ranging from surface skimming to deep plunges—all these bird species share a common ecological strategy. They exploit prey concentrated by pelagic predators within marine predator–fish associations, demonstrating how varied adaptations can support similar feeding opportunities in marine ecosystems.

## 8. Multi-predator feeding aggregations: who does what?

In pelagic habitats, large predators employ a remarkable diversity of strategies to capture prey, reflecting the complex physical and ecological constraints of open water environments. These strategies extend beyond simple attack-capture sequences, encompassing endurance-based exhaustion of prey, group hunting to concentrate prey and the use of specialized body weapons such as bills or tail slaps to incapacitate or kill prey (see figure 2). Beyond these mechanisms, other morphological and behavioural adaptations play critical roles. For example, a stealthy approach, which reduces the flight initiation distance of the prey, can increase hunting success, especially in open water where concealment options are limited [19]. Similarly, the evolution of control surfaces, including fins, flukes and tail morphology, allows large predators to maintain manoeuvrability relative to smaller, highly agile prey, mitigating one of the fundamental challenges of hunting in pelagic systems ([12,53]; figure 2). Taken together, the wide range of locomotor, physiological and behavioural traits observed among pelagic predators supports the idea that predator assemblages are structured by trait-mediated role differentiation, with different species contributing to distinct stages of the hunt (points (v) and (vii)). Under such conditions, predators may occupy complementary niches that enhance the overall efficiency of multispecies predation events.

By definition, MPFAs involve interactions among species, which create opportunities for coordinated hunting, particularly among taxa that benefit from collective strategies. Smaller predators may be more prone to group hunting at MPFAs, as they require a smaller proportion of the prey, are less likely to interfere with each other and may gain protective advantages against larger predators. Morphology and hunting strategies may further influence interspecific dynamics: species capable of aggregating in large numbers could dominate resource access, potentially swamping solitary or less abundant predators [66,108]. The behaviour of one species can have cascading effects on others; for instance, herding prey may facilitate lunge feeders while impeding predators that rely on isolating individuals, though predators may also attempt isolating the prey [75]. This dual effect highlights the context-dependent balance between facilitation and competition within MPFAs (point (iv)), as well as the potential for conflicting prey-state preferences among predator types (point (v)). In the future, the use of biologging tags (with mini cameras) may help elucidate if a given predator species changes its attack kinematics significantly in the presence of heterospecific predators, indicating a potentially costly relationship within the MPFA.

Detailed field studies provide compelling evidence for a phased-approach to predation. In a previous study off the Azores, Clua & Grosvalet examined interactions among dolphins, large tunas and seabirds feeding on schooling fish [3]. They identified four distinct phases of MPFAs: a preparation phase, an intensification phase, a mature phase and a dispersion phase. These phases appear generalizable across many pelagic systems albeit with different species that have similar ecological roles to the ones observed in Clua & Grosvalet's study. During the preparation phase, group hunting dolphins locate prey patches at depth, sometimes down to 25 m, attracting seabirds to the feeding area. This initial search and concentration of prey is critical, as prey are often patchily distributed. The intensification phase occurs when prey are herded towards the surface, facilitating easier access for multiple predator types. This provides direct support for the hypothesis that herding is a collective process that enhances prey accessibility (point (i)). Dolphins begin feeding during this phase, employing coordinated movements to maintain prey in a concentrated ball. The mature phase is characterized by the arrival of additional predators, such as large tunas and seabirds, which exploit the optimally concentrated prey patch. In other systems, predators with large engulfment capacities, such as baleen whales, are able to capitalize on prey patches aggregated by early arrivals of predators [88]. Similarly, predators equipped with specialized weapons, such as billfishes, can access prey that may otherwise escape smaller predators. Comparable successions occur in other ecosystems; for example, sea lions exploit schooling fish driven to the surface by the high-speed manoeuvres of striped marlin [64]. Finally, the dispersion phase marks the end of foraging, often when repeated high-speed attacks, for example by ramming schools of tunas [3] or baleen whales [88], fragment the prey school, dispersing the previously aggregated prey. This stage corresponds to the disruption phase, where repeated or simultaneous attacks reduce prey cohesion and escape success (point (ii)).

Throughout the hunts observed, various birds were present, especially Cory's shearwater (*Calonectris diomedea borealis*) which did not dive beyond 2 m to pursue their prey [3]. There are a number of other examples of aquatic predators that drive their prey to the surface, which provides foraging opportunities for birds [1,109,110]. Recent work based on drone footage from Malaysia specifically showed that terns (*Onychoprion* sp.) tend to attack when schools of anchovies (*Encrasicholina* sp.) and sardinellas (*Amblygaster* sp.) are less cohesive as a result of sailfish strikes [100]. This suggests that sailfish indirectly help terns by making prey easier to catch, supporting the idea that the lower prey school cohesion caused by sailfish can benefit seabirds.

Across these systems, a common pattern emerges: the initial effort by one species to concentrate prey creates opportunities for subsequent arrivals of other predator species. This sequence exemplifies how facilitative interactions emerge from trait differences among predators, with early-arriving species effectively engineering prey availability for others (point (iv)). These benefits manifest in several ways: (1) prey become more accessible near the surface, (2) prey fatigue increases through sustained herding by the predators, and (3) injuries inflicted by predators using physical weapons reduce prey escape capacity. In turn, these injured or fatigued individuals provide profitable opportunities for slower or less agile predators that might otherwise struggle to capture healthy, evasive prey. However, these dynamics also introduce potential for conflict. When trait differences allow certain species to systematically exploit the hunting efforts of others, kleptoparasitism and interference competition can dominate interactions. For instance, humpback whales may appropriate bait balls created by killer whales [88,111], while sea lions joining the foraging areas of striped marlin can reduce the marlins’ capture success [64]. Such interactions are predicted to be strongest when predators share similar functional roles or target similar prey states, providing a basis for identifying trait combinations that generate competitive interference (point (vi)).

Ultimately, the interplay of locomotor capabilities, sensory performance and behavioural strategies determines each species’ role at any given phase of the hunt. Some predators are adapted for long distance swimming and high frequency of prey encounters of prey patches, while others specialize in high-speed pursuits or precise manoeuvres during the terminal stages of capture. Trait-mediated role differentiation probably enhances the overall efficiency of predation in multispecies events, allowing multiple predators to exploit the same prey resource while reducing direct interference. Taken together, these patterns suggest that predator morphology and physiology jointly determine both participation in hunting stages (point (vii)) and the balance between competition and facilitation within MPFAs (points (iv) and (vi)). At the same time, these interactions generate shifting opportunities for competition, exploitation and dynamic hierarchies, creating a highly flexible and context-dependent framework for predator–prey dynamics in pelagic systems (figure 3). Understanding these processes can not only help identify the ecological roles of individual species but also provide insight into how complex behavioural and physiological traits have co-evolved in response to the spatial and temporal challenges of open-ocean foraging.

**Figure 3.**
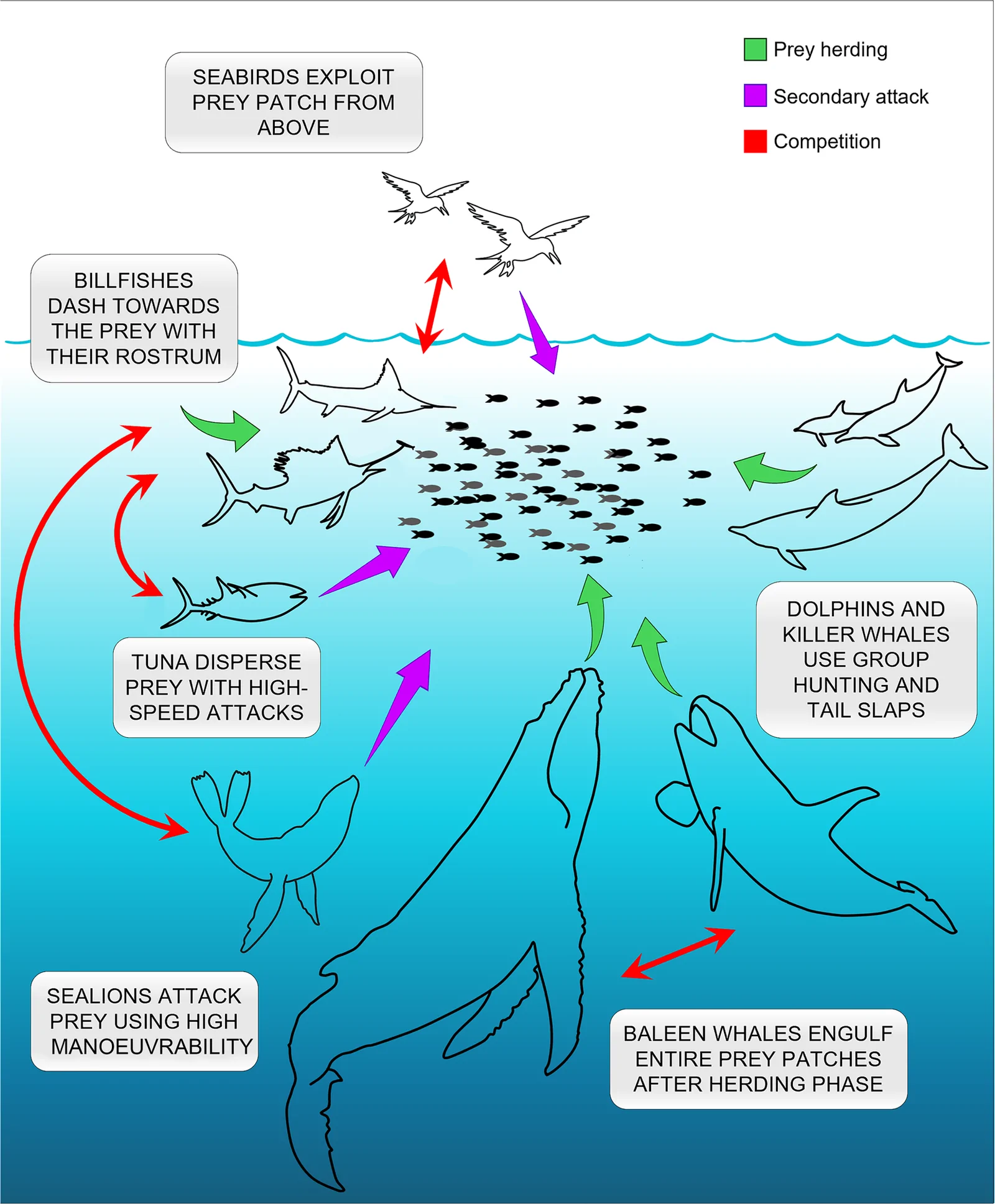
General scheme for the different attack strategies of the main taxa discussed here. Prey herding, secondary attacks and potential competition are identified. Only some examples of competition are identified. See text for more details.

## 9. Conclusions

In pelagic settings, schooling prey are frequently targeted by diverse assemblages of predators, creating MPFAs in which species with markedly different traits interact around the same resource (figure 3). This diversity in speed, manoeuvrability, sensory capabilities, social behaviour and prey-handling tools is expected to shape not only each species’ hunting strategy but also their relative performance, roles across different stages of the hunt and the balance between competition and facilitation within an aggregation (points (iii), (iv) and (vii)). In particular, collective processes, such as prey herding and disruption, are likely to emerge from the coordinated or cumulative actions of multiple predators, especially where individual performance is constrained (points (i) and (ii)), while differences in morphology and behaviour may promote either niche partitioning or, alternatively, competitive interference depending on the degree of functional overlap among species (points (v) and (vi)). Identifying the performance limits and functional tools available to each predator species is therefore fundamental for understanding the mechanisms that underlie their relative success, coexistence and potential interspecific competition. Research that includes analysis of the behaviour of the prey, and whether or not it shows predator-specific defensive strategies, would be an important further step towards understanding how predator traits and collective behaviours shape prey responses and, ultimately, the outcome of predator–prey interactions in pelagic systems. Such work will ultimately deepen our understanding of the ecological and evolutionary processes governing MPFAs, providing a predictive framework for how trait-mediated interactions structure predator assemblages and determine ecosystem-level dynamics under changing environmental conditions.

## Data Availability

This article has no additional data.

## Theme

One contribution of 14 to a theme issue ‘Multi-predator feeding aggregations in pelagic waters’.
